# Smart Materials

**DOI:** 10.3390/ma15186307

**Published:** 2022-09-11

**Authors:** Luigi Fortuna, Arturo Buscarino

**Affiliations:** 1 Dipartimento di Ingegneria Elettrica Elettronica e Informatica, University of Catania, 95125 Catania, Italy; 2 IASI, Consiglio Nazionale delle Ricerche (CNR), 00185 Roma, Italy

The future of engineering systems is based on the capability of integrating sensors, actuators, control systems and materials. Engineering products, in fact, are nowadays innovative only if they ensure safety, reliability and good quality of life in all parts of the world. New materials, therefore, must be as much as possible *green* and smart. The capability of smart materials to adapt themselves to the environment and to the various system conditions are fundamental in the future. Moreover, the possibility of using control signals to modify the properties of the materials provides the real possibility to design innovative systems with a wide range of applications.

All these innovative concepts living in smart materials rely on having accurate mathematical models. Moreover, the design of materials requires appropriate software tools and advanced sustainable technological processes to produce them. In order to have both reliable and smart materials, more validation experiments are required for their complex characterization.

The topic of smart materials is linked to a strongly interdisciplinary area of research. Moreover, the global smart materials market size has been about 60 billion USD in 2021 and it is expected to register an increase of more than 13% within the next two years. The impressive impact that smart materials had in the years 2020–2021 in the journal Materials motivates this Editorial. A limited number of 15 papers have been selected for this aim and constitute the backbone of this Editorial.

The Editorial is organized as follows. At first, a glossary summarizing the particular material types is reported. A short description of the 15 selected papers will follow. The Editorial will be concluded with a summary Table indicating the various correlations among the 15 papers and with a final consideration.

**Glossary:**

**Magnetorheological Fluids (MRF)** are considered smart fluids. The application of a magnetic field allows controlling their viscosity. Made by a carrier oil and magnetic particles are different from ferro-fluidics where the particles are of smaller size.

**Shape Memory Alloy (SMA**) materials, such as TiNi composite, have thermoelastic martensive transportation characteristics upon heating. The shape recovery occurs at a transformation temperature called austenitization temperature.

**Composite Materials (CO)** are made by two or more materials. The material could have different properties from chemistry-physical characteristics; moreover, the composite has different peculiarities with respect to its components.

**Elastometers** are polymers with elastic properties including components with weak or strong links.

**Metamaterials** are artificially structured materials that have the capabilities to control electromagnetic waves, acoustic waves and other types of waves, and can realize negative refraction indices.

In the paper, [1], Wang and co-authors present an accurate and efficient mathematical model of a composite device made by a rod with two piezoelectric layers CoFe${}_{2}$O${}_{4}$ (or Terfenol-D) and one made by semiconductor n-Type ZnO. The analytical solution of the partial differential equation is reported. It is, moreover, shown that the application of an axial magnetic field produces some physical effects, including extensional deformations and redistribution of mobile charges in the semiconductor. The analytically proved results allow designing an optimal geometrical structure to get effects making the composite rod an efficient magnetic field sensor or a magnetic field-to-field transducer. The presented model is conceived for 1D structures but could be extended to 3D configurations.

The contribution presented by Zhang et al. in [2] is related to the mathematical modeling of an MRF. The key-point of the proposed approach consists of modeling the Carbon Iron Powder (CIP) in terms of interacting magnetic units that allow the characterization of a collective motion behavior described by a Newtonian kinetic equation. Due to the importance of the particles in MRFs, the simple but accurate model of each unit allows estimating with accuracy the behavior of the smart MRF.

In the research presented in [3] Yoon and co-authors present a study that referred to materials conceived as composite and related to MRF. Moreover, the materials that are used for the composite are also MRF. In particular, magnetorheological elastomers (MRE) and MRF are considered. The paper is finalized to the realization of smart materials and also to characterize, from a mechanical point of view, soft materials with adaptability performances. The paper presents more results concerning the aspect of the control action of magnetic fields in terms of displacement force behavior.

The survey presented in the paper by Vermes and Czigany [4] reviews non-conventional deformations derived by various types of actuators with different actuation mechanisms. The paper covers the different strategies that can be adopted for shape adaptation, like light-pressure and chemical shape adaptation, mechanical-actuated shape adaptation and electro-actuated systems. The paper reviews in detail and in a clear manner also non-conventional types of actuators by using very exhaustive tables including for each actuator the common details, including the working principles, the deforming material structure, the deformation mode, the adopted testing strategies, the mathematical model approach, the comments about the specific results and performances. The paper covers almost all items related to the smart material topic.

The SMA materials have been considered one of the more appealing classes of smart materials. Even if the various proposed applications and the various characterization approaches had a wide diffusion for SMA material, efficient and reliable methods to define sensitivity models and uncertainty have not yet been satisfactorily studied. In the contribution [5] Islam and Karadoǧan, based on the Ishin-Perce model, developed more Matlab functions in order to have a tool allowing both the characterization and the design of SMAs. The numerical simulations are referred to more physical parameters and to more operating conditions (temperature, loading). Moreover, some results, based on a Monte-Carlo simulation approach, are presented. A detailed discussion based both on physical considerations and numerical analysis is performed.

The importance of composite materials and devices based on either classical components or on SMA is remarked in the contribution [6] by Hamann and co-authors. The paper shows an outstanding application in the area of orthopedical implants. It is shown that nitinol-based implants can allow a better functionalization in repairing human cell structure with respect to the classical implants, being the degree of self-adaptability of the SMA components responsible for it. The study addressed two main points. The first one is devoted to the accurate 3D printing realization of the composite repair element, while the second regards the accurate biological and clinical data analysis in order to validate the approach and increase the anchorage stability and osteointegration.

The behavior of SMAs is strongly related to martensite transformation. Ju and co-authors [7] consider complex SMAs based on Ni-Mn-Ga-Co-Gd. This leads to the so-called high-entropy design based on martensitic transformation. This allows having SMAs with high martensitic transformation temperatures, in the order of 500 °C, with quite improved behavior. The study includes also the characterization of the martensitic transition. The performed study is highly experimental and the results have been validated on the analysis of microstructured patterns obtained by scanning electron microscopy images. The research is mainly addressed to validating complex microstructures.

The property of smart materials can also be obtained by modifying flat textiles with hydrogels (hydrophilic polymers that are not dissolved by water). The property of hydrogels is very interesting as regards both the mechanical adaptability and the ease of use in biomedical applications, like contact lenses. The study presented by Sa̧siadek in [8] is referred to the characterization of hydrogels-textile compounds that are sensitive to UV radiations. Therefore, it is conceived as a textile material with the integration of Pluronic F-127 and NBT allowing an accurate characterization and response to a wide range of radiations and not only UV.

SMAs with a sustainable structure can be also conceived for micro and nanoscale applications. Bhale et al. [9] characterize SMAs based on Ti${}_{2}$NiCu. The experimental study presented in the contribution opens a new possibility for using high-performing SMAs, as regards electrocaloric effects, shape memory and superelasticity properties in nanoscale applications, also in the area of biomedicine.

The mathematical model of innovative materials represents a fundamental step for the validation of their smart properties. It is considered outstanding, in this context, the contribution by Wang et al. [10]. The authors propose an SMA mathematical model in terms of the temperature dependence, the tensile-compressive asymmetry and the ratcheting effects of the material. The proposed model can be handled in an easy manner and the analytical solutions of the PDEs are presented. Even if the SMA material is not specified (it is supposed to be a classical one), the experimental trends show the very good quality of the mathematical model. Appealing results are also shown in terms of numbers of tensile-compressive cycles.

The contribution proposed in [11] by Ma et al. regards a smart class of elastomers, called Dielectric Elastometer Actuators (DEAs). In the proposed scenario the actuators are based on two compliant electrodes (CEs). Their characteristics are high conductivity, and low elasticity with adaptability properties. The accurate characterization and the physical morphological structure of CEs are included in the paper, as well as strategies in order to obtain the best performances for the DEA system. In the paper, the possibility of using the DEA system as an actuator in robotics and in biomechanical applications is also discussed.

Smart materials are also involved in optical and radiofrequency applications. In the contribution by Mou et al. [12], the authors propose metamaterial-based smart systems with tunable multiband and ultraband possibilities, realized in vanadium dioxide. The authors propose both models and experiments that show temperature-controlled tunable materials. The paper opens a new area of research on smart material for high-frequency applications. The included experimental results are promising.

The contribution presented by Rogóż et al. in [13] addresses the problem of realizing linear motors by properly configuring liquid elastomers. The liquid crystal elastomers are controlled by laser light impulses allowing technically the deformation of the elastomer which is converted to a propulsive force. Accurate measurements and various experiments allow validating the efficiency of the system.

Future advanced applications of smart materials related to MEMS and Microsystems, like microstents or microgrippers need further investigations. Jiao et al. in [14] investigate a class of mechanical metamaterials (not to be confused with optical metamaterials) that can be terminally controlled. The geometrical structure is fundamental in the behavior of these systems. The paper includes more mechanical structures and the use of numerical tools to simulate both the mechanical movements and the thermal state of the various structures. Even if a detailed mathematical model of the thermo-mechanical system is presented, it is not indicated the heating system.

The contribution by Muhazeli et al. in [15] presents devices based on MRF controlled by magnetic fields with the aim of proposing tunable noise absorbers, in a wide range of acoustical frequencies. The paper shows in detail their peculiarities and how to build the devices. The experimental results coupled with a signal processing procedure show the efficient applicability of the proposed smart system.

In Table 1, the correlations among the various papers and key topics are presented in order to give the readers a compact and complete scenario of the related subjects.

In addition, the Editorial aims at remarking some details of further papers, in order to reinforce some items on the subject.

In particular, in the papers by Roupec et al. [16], and Muhammad Zaki et al. [17], further efforts are given toward MRF materials. In [16], a detailed comparison among more MRF is reported, while in [17] MRF based on Petroleum oils is widely studied. The nano-scale applications of smart materials are studied also by Bormashenko et al. in [18], while the topic of UV smart fibers is also remarked in the paper by Sa̧siadek et al. [19]. The paradigm of smart materials is presented both in [20] by Li et al. and in [21] by Nie and co-authors, where the application to large space civil structures is presented for rail systems monitoring. The possibility of modeling piezoelectric devices based on artificial neural networks is presented in [22]. This paper, by Min et al, opens an innovative way for smart material modeling. The topic of smart materials covers also accurate surface technology for electrode processing, this is the subject of the paper by Svetlizky et al. [23].

These last items allow the reader to have a complete overview of recent papers on smart materials published in the journal Materials, further outlining the multidisciplinarity of the topic and the multi-technological challenges in different system scales, from nanoscales to the very large integration scales.

## Figures and Tables

**Table 1:** Correlation matrix of the selected key topics.

	**[1]**	**[2]**	**[3]**	**[4]**	**[5]**	**[6]**	**[7]**	**[8]**	**[9]**	**[10]**	**[11]**	**[12]**	**[13]**	**[14]**	**[15]**
Composite materials	X		X	X				X			X				
SMAs				X	X	X	X		X	X					
MRFs		X	X												X
Material math. modeling	X	X		X	X	X	X			X		X		X	X
Experimental platforms		X	X	X		X	X	X	X	X	X	X			X
Engineering appl.	X	X	X	X		X	X	X	X	X	X	X	X	X	X
Medical appl.				X		X		X			X				
Robotics				X					X		X		X	X	X
Actuators	X			X					X		X		X		X
Sensors	X			X				X	X			X	X		
Dev. of software tools		X			X	X							X	X	
Scale							Micro Nano		Nano					Micro	Medium
Elastometers				X							X		X		
Control	X	X	X	X	X	X	X	X	X	X	X	X	X	X	
